# Herpetic Pneumonia in Indian Ringneck Parrots (*Psittacula krameri*): First Report of Novel Psittacid Alphaherpesvirus-5 Infection in Europe

**DOI:** 10.3390/ani12020188

**Published:** 2022-01-13

**Authors:** Marco Bottinelli, Andrea Fortin, Claudia Zanardello, Jane Budai, Federica Gobbo, Gianmaria Antonazzo, Stefania Leone, Marianna Merenda, Calogero Terregino, Salvatore Catania

**Affiliations:** 1 SCT1-Verona, Istituto Zooprofilattico Sperimentale delle Venezie, 37060 Buttapietra, VR, Italy; sleone@izsvenezie.it (S.L.); mmerenda@izsvenezie.it (M.M.); scatania@izsvenezie.it (S.C.); 2 SCS6 Virologia Speciale e Sperimentazione, Istituto Zooprofilattico Sperimentale delle Venezie, 35020 Legnaro, PD, Italy; afortin@izsvenezie.it (A.F.); jbudai@izsvenezie.it (J.B.); fgobbo@izsvenezie.it (F.G.); cterregino@izsvenezie.it (C.T.); 3 SCS3 Diagnostica Specialistica e Istopatologia, Istituto Zooprofilattico Sperimentale delle Venezie, 35020 Legnaro, PD, Italy; czanardello@izsvenezie.it; 4 Private Practitioner—Ambulatorio Veterinario Vestina, 65015 Montesilvano, PE, Italy; info@ambulatoriovestina.it

**Keywords:** herpesvirus, PsHV-5, parrots, respiratory disease, pneumonia

## Abstract

**Simple Summary:**

*Herpesviridae* is a large family of double-stranded DNA viruses that infect many different animal species. Herpesvirus infections are common in avian species and, to date, three different alphaherpesvirus species have been recognized as causative agents of disease in Psittaciformes. However, there are reports of respiratory disease in parrots characterized by the presence of distinctive herpes-related histologic lesions, albeit with no identified etiology. Our study acknowledges the unprecedented presence of the novel *Psittacid alphaherpesvirus-5* in Europe. Necropsy was performed on Indian ringneck parrots deceased after severe respiratory distress and diagnosis was achieved through histological examination, visualization of the virions by electron microscopy and genome sequencing. The pathogen has been reported only once in Australia and the present report raises the probability that its distribution is wider and it should be included in the list of pathogens threatening parrot populations.

**Abstract:**

The first two European outbreaks of herpetic pneumonia caused by *Psittacid alphaherpesvirus-5* were diagnosed based on gross pathology findings, histological examination, transmission electron microscopy visualization and genome sequencing. The outbreaks, characterized by high morbidity and high mortality rates, involved two parrot species, namely the Indian ringneck parrot (*Psittacula krameri*) and the Alexandrine parakeet (*Psittacula eupatria*). Clinical signs observed were ruffled feathers, dyspnea, tail bobbing, open wings while breathing, depression and anorexia. Necropsy was performed on Indian ringneck parrots only, and the most evident and serious gross lesion found in all the birds was a diffuse marked consolidation of the lungs associated with parenchyma congestion and oedema. Histological examination confirmed the existence of bronchopneumonia characterized by the presence of syncytial cells with intranuclear inclusion bodies. In one bird, fibrinous airsacculitis was observed as well. Lung tissue inspection through electron microscopy revealed the presence of virus particles resembling herpesviruses. Viral DNA was extracted, amplified using primers for *Alloherpesviridae* DNA polymerase gene detection, and then sequenced. BLAST analysis showed a 100% identity with the only previously reported sequence of PsHV-5 (MK955929.1).

## 1. Introduction

Avian veterinarians are aware that most of the critical avian patients are brought to visit with respiratory distress [1,2]. Classical clinical signs are tachypnea, dyspnea, mouth breathing, voice change and exercise intolerance, which are usually accompanied by general signs of illness. Different infectious agents, such as parasites, bacteria, fungi and viruses, can cause disease of avian upper and lower respiratory tracts [3].

Viruses belonging to the *Herpesviridae* family make up one of the most important groups of viruses due to their ubiquitous nature, evolutionary diversification, and their role in serious medical and veterinary diseases [4]. Avian herpesviruses may infect different hosts and usually show a tropism for epithelial cells and both lymphatic and nervous systems. Their virulence varies widely and it is known that they usually cause a mild disease in their natural hosts; however, it has been speculated that, when cross-species infection occurs, the resulting disease may become severe and fatal [5,6]. In addition, these viruses are able to establish latent infections, surviving from generation to generation since they are periodically reactivated and shed, making it hard to contain their spread in the susceptible population [7,8].

Of the nineteen avian herpesviruses identified so far, three alphaherpesviruses have been found in parrots as cause of disease: *Psittacid alphaherpesvirus-1* (PsHV-1, causative agent of Pacheco’s Disease) [9], PsHV-2 [10] and PsHV-3 [11]. *Psittacid alphaherpesvirus-1* and PsHV-2 are closely related to each other and have the ability to induce the development of mucosal papilloma [12,13]. In addition, PsHV-1 is either able to cause an acute, fatal hepatic necrosis, or to induce neoplastic lesions of both bile and pancreatic ducts long after they have first encountered their host [14]. In contrast, PsHV-3 has been shown to have a specific tropism for the respiratory tract in different avian species [11,15]. Recently, a novel avian alphaherpesvirus, named *Psittacid alphaherpesvirus-5* (PsHV-5), has been detected for the first time in Australia from Indian ringneck parrots (*Psittacula krameri*) showing severe respiratory disease [16]. Viral genome sequencing revealed that PsHV-5 is related to PsHV-3 and it is quite distant from other avian herpesviruses to the point that it can be considered a distinct virus species within the genus *Iltovirus* [16]. However, information on its global distribution, host range and clinical/pathological presentation of PsHV-5 infection are scarce and refer to a single confirmed case.

Since there are no other reports available on the major scientific databases, we document the first detection in Europe of PsHV-5 during two independent outbreaks involving two psittacine bird species (*Psittacula krameri* and *Psittacula eupatria*). Thus, the aim of the authors is to transfer additional information on PsHV-5 infection to avian veterinarians, parrot breeders and the broader scientific community, expanding the knowledge on the epidemiology, the clinical–pathological appearance of this new viral infection in parrots, as well as on the morphologic and genetic features of the virus.

## 2. Materials and Methods

### 2.1. Case Description and Diagnostic Investigations

Two outbreaks of severe respiratory disease were observed between July and August 2021 in two different aviary flocks located in geographically distant Italian regions. The first outbreak affected a flock that consisted of ten young (less than 1 year of age) Indian ringneck parrots bought in block from a specialist pet dealer. The second one broke out after an Alexandrine parakeet (*Psittacula eupatria*) and seven Indian ringneck parrots, that came from a pet dealer, had been introduced into a private collection of ornamental birds, where other Indian ringneck parrots were also present. Two Indian ringneck parrots from the two different affected flocks were submitted to the diagnostic facility of the Istituto Zooprofilattico Sperimentale delle Venezie (IZSVe) for post-mortem examination. Tissue samples were collected for bacterial culture, transmission electron microscopy and histological assessment. A third Indian ringneck parrot coming from the second outbreak was conferred at a later date and was analyzed as well.

### 2.2. Bacterial Culturing

Aseptically collected lung and brain tissue samples were cultured on Blood Agar (Agar base (Biolife Italiana, Milan, Italy), sheep blood (Allevamento Blood, Castellalto, Italy)), MacConkey’s Agar (Biolife Italiana, Milan, Italy) and Bile-Esculine Agar plates (Biolife italiana, Milan, Italy). The plates were incubated at 37 ± 1 °C under controlled aerobic atmosphere as well as 5% CO_2_ atmosphere. Plates were checked for microbial growth at 24 and 48 hours post-incubation. In order to exclude the presence of bacterial growth inhibition, a tissue aliquot of each organ was placed inside a tube containing enrichment broth (heart infusion broth) (Biolife italiana, Milan, Italy) and incubated at 37 ± 1 °C under controlled aerobic atmosphere for 24 hours. A 10 µL aliquot was then used for bacterial culture following the same passages described above.

### 2.3. Histopathological Examination

Lung samples from all the three birds were sent to the IZSVe histopathology laboratory (SCS3 Diagnostica specialistica e istopatologia). Based on the available literature and gross-pathology findings, it was deemed necessary to ask for and analyze samples from the trachea, air sac, liver and heart of the third bird. All the samples were fixed in 10% buffered formalin, routinely processed for histology and 4 μm sections were stained with hematoxylin and eosin.

### 2.4. Transmission Electron Microscopy

For the detection of viral particles by means of negative staining electron microscopy [17,18,19], a homogenate was obtained by grinding 1 g of lung tissue sample in 5 mL of sterile distilled water (1:5 *w*/*v*) using a small mortar and pestle with the aid of fine sterile quartz. Aliquots of lung tissue from the three parrots were processed, as well as liver and brain tissue samples from the third bird. The homogenate was first frozen at −20 °C, thawed in a water bath at 37 °C for 15′, and then centrifuged at 3500 rpm or 2205× *g* for 15′ and the supernatant at 15,000 rpm or 24,133× *g* for 15′ for clarification. Once 90 µL of the second supernatant were vialed, a formvar/carbon-supported copper grid (Formvar/Carbon Copper Grid 200 Mesh, Electron Microscopy Sciences, Hatfield, PA, USA) was placed flat on the bottom of the vial. This preparation was spun at high speed (28–30 psi at 95,000 rpm or 100,000× *g*) for 15′ (Airfuge Air-Driven Ultracentrifuge, Beckman Coulter Life Sciences, Indianapolis, IN, USA). The grid was stained with 10 µL of 2% phosphotungstic acid (PTA) (pH 7); the PTA was left on the grid for a few seconds (8–10 s). The grid was then examined under an EM 208S transmission electron microscope (Philips, Amsterdam, Netherlands) and virus particles were observed with Megaview III and measured using iTEM software (Olympus SIS, Münster, Germany).

### 2.5. DNA Extraction and Sequencing

DNA extraction was performed on lung tissue samples from all the three parrots. The total DNA was purified from 300 µL of sample suspension using the QIAsymphony^®^ DSP Virus/Pathogen Midi Kit (Qiagen, Hilden, Germany) on a QIAsymphony^®^ SP instrument (Qiagen, Hilden, Germany). Viral genome detection was carried out using previously published primers, designed to detect the genome region that codifies for the most conserved domain of *Alloherpesviridae* DNA polymerase gene [20], and Platinum Taq DNA Polymerase Kit (Invitrogen, Waltham, MA, USA). One µM of each primer and 5 µL of template DNA, in a final volume of 25 µL, were used. Runs were performed on a C1000 Touch Thermal Cycler (Biorad, Hercules, CA, USA) under the following cycling conditions: 95 °C for 5′, followed by 30 cycles at 95 °C for 30′, 51 °C for 30′, 72 °C for 2′ and a final elongation of 72 °C for 5′. Proper amplification was conducted using the QIAxcel Advanced System (Qiagen, Hilden, Germany) capillary electrophoresis system. The Sanger method was used to sequence the 495 bp product. The resulting sequence was aligned against the integrated NT database using BLAST [21]. In order to verify the accuracy of the attribution, a phylogenetic tree was built employing the nucleotide sequences of the DNA polymerase gene of 20 avian herpesviruses.

## 3. Results

### 3.1. Outbreak Information

In the first case, the animals had started to show clinical signs of respiratory distress one day after arriving at the owner’s house. Over time, clinical signs such as ruffled feathers, dyspnea, tail bobbing and open wings while breathing became more and more severe until death. At the end of the observation period, the morbidity rate was 100% and half of the animals (5/10) had died. The remaining birds were finally returned to the seller.

In the second case, the Alexandrine parakeet and three of the seven Indian ringneck parrots started to show increasingly severe respiratory distress 20 days after their introduction into the private bird collection. The Alexandrine parakeet was not put into quarantine and was placed with a female bird of the same species in a dedicated cage, within a battery cage housing system consisting of 25 cages located in an outdoor setting. The Indian ringneck parrots were in the adjacent cage. Clinically, in addition to the signs described above, a marked sense of depression and anorexia were observed. Later, two out of the three sick Indian ringneck parrots died, while the symptomatic Alexandrine parakeet completely recovered from illness. The female Alexandrine parakeet and the four other Indian ringneck parrots never developed any signs of respiratory disease.

### 3.2. Gross Necropsy Findings

**Figure 1:**
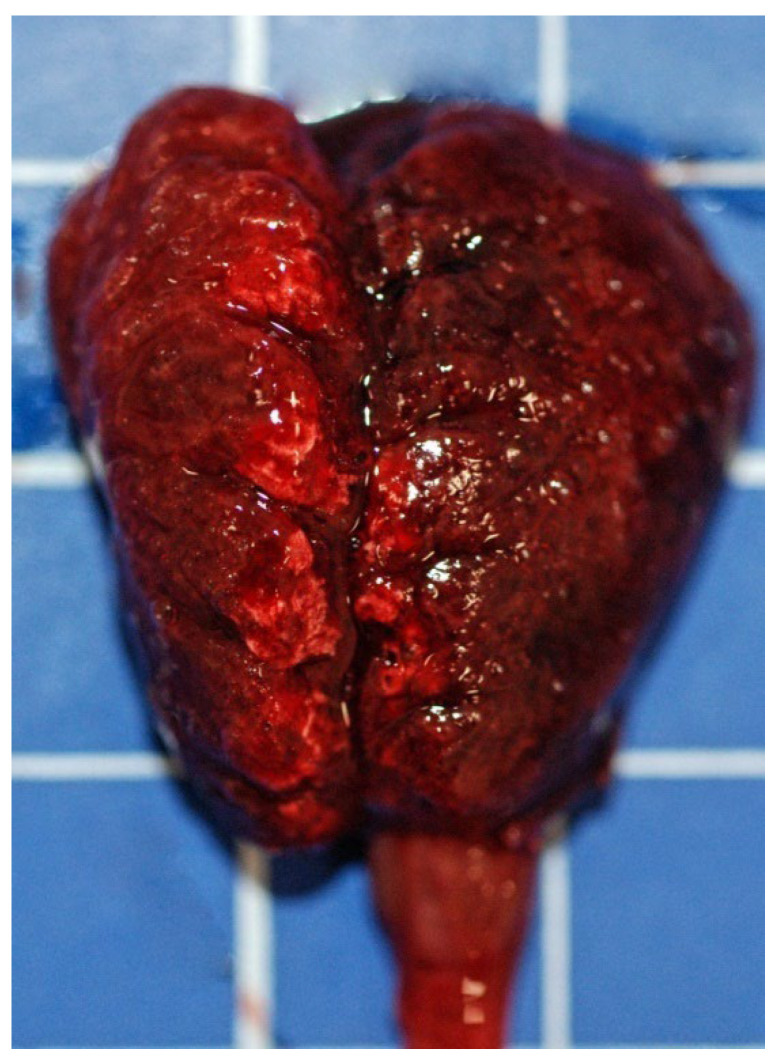
Gross photo of an Indian ringneck parrot’s lungs showing diffuse marked consolidation and congestion. Square = 1 cm.

External examination revealed poor (case 1) to almost good (case 2) nutritional status and feces around the cloaca in both birds. No other feather-related abnormalities were observed. At the inspection of the coelomic cavity, diffuse marked consolidation and congestion of the lungs, which were also edematous (Figure 1), together with mild hepatomegaly were observed in both cases. In case 1, mild foamy airsacculitis and a diffuse moderate congestion of the epicardium were also observed. Macroscopically, there was no presence of any type of material in the lumen of the pharynx, trachea and larynx of both birds. No food material was found in the crop of the animals inspected. In addition to the abovementioned lung lesions, the third bird (case 2) showed a marked fibrinous airsacculitis and a marked diffuse pericardial congestion, as well as mild hepatomegaly and moderate enteritis with dilatation of the duodenal loop.

### 3.3. Microbiology

No bacterial growth was observed from any of the tissue samples.

### 3.4. Histopathological Findings

**Figure 2:**
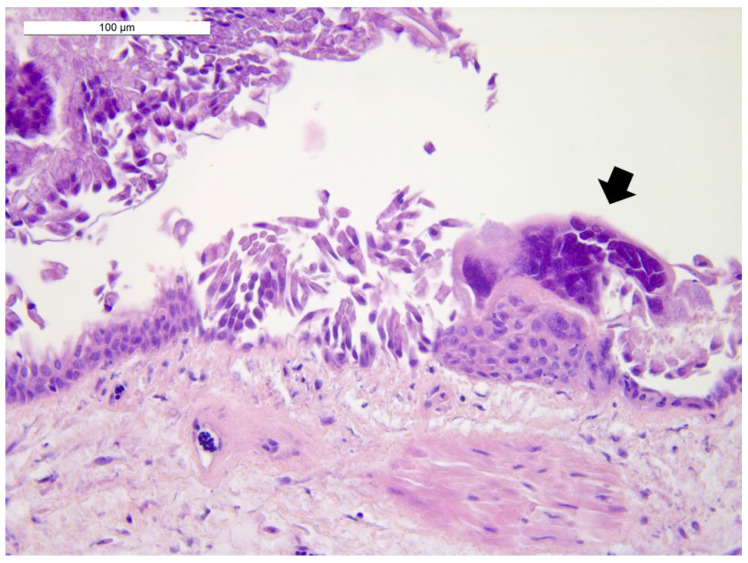
Lung: a syncytial cell (arrow) with intranuclear eosinophilic inclusion bodies. Hematoxylin and eosin (40× objective). Bar = 100 µm.

**Figure 3:**
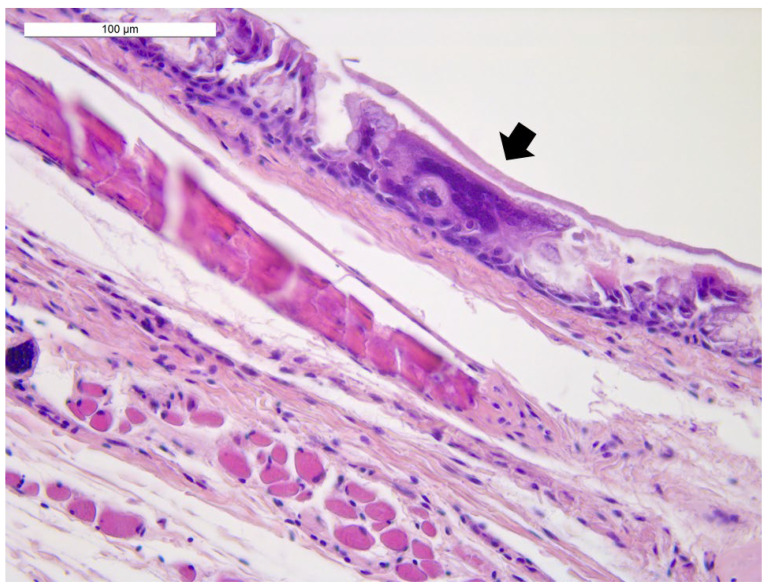
Trachea: necrosis and desquamation of epithelial cells with the presence of syncytial multinucleated cells and superficial muco-fibrinous material (arrow). Hematoxylin and eosin (40× objective). Bar = 100 µm.

**Figure 4:**
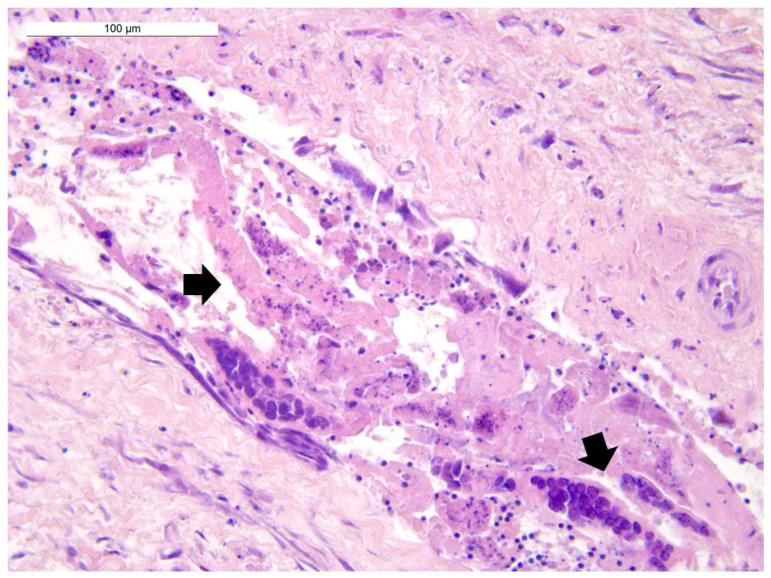
Air sac: fibrinous exudate and necrotic debris admixed with multinucleated syncytial cells and mild heterophilic infiltration in the air sac lumen (arrow). Hematoxylin and eosin (4× objective). Bar = 100 µm.

Histological examination revealed diffuse autolytic phenomena in the lung samples from the first two birds, resulting in difficult morphological evaluation of the parenchyma. Nevertheless, a moderate congestion of the parenchyma was observed, as well as the presence of luminal fibrinous exudate in bronchi and parabronchi with epithelial necrosis and syncytial cells with intranuclear inclusion bodies (Figure 2). The lung sample from the third bird showed mild and multifocal respiratory epithelium hyperplasia with large syncytial cells with intranuclear eosinophilic inclusion bodies. Moderate fibrinous heterophilic bronchopneumonia and parenchyma congestion were detected. The third parrot’s trachea, air sac, liver and heart were also examined. The trachea showed necrosis and desquamation of the epithelium with the presence of syncytial multinucleated cells with eosinophilic intranuclear inclusion bodies. A mild mononuclear inflammatory infiltrate in the lamina propria and the presence of fibrinous material in the lumen were also detected (Figure 3). The air sac was thickened and showed a hyperplastic epithelium with syncytial multinucleated cells with inclusion bodies. Fibrinous exudate and inflammatory, mostly heterophilic infiltration were also observed (Figure 4).

No significant histological lesions were detected in the heart and liver samples. A histopathological diagnosis of viral pneumonia, tracheitis and fibrinous heterophilic airsacculitis caused by herpesvirus was made.

### 3.5. Electron Microscopic Findings

**Figure 5:**
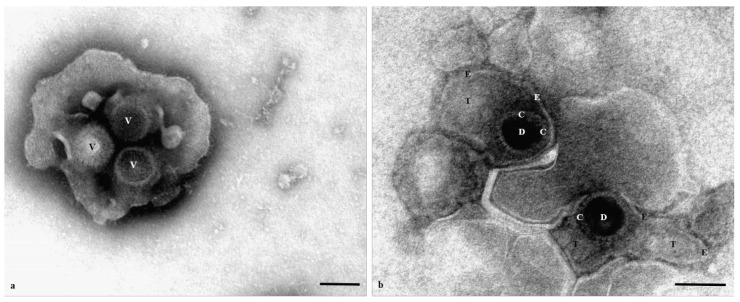
Negative-staining electron microscopy micrographs of herpesvirus particles (at 71k× magnifications). Bars = 100 nm. (**a**) Three non-enveloped virions (**V**) showing icosahedral nucleocapsid with distinct capsomers. Take note of the stain penetrated in the central hole of the capsomers. (**b**) Two enveloped particles with stain penetrated nucleocapsids. Take note of the representative four-layered structure of the virions: Core (DNA genome) (**D**); Capsid (**C**); Tegument (**T**) and Envelope (**E**).

Negative-staining electron microscopy of the lung homogenates revealed the presence of numerous virus particles, single or in small groups, morphologically resembling herpesviruses (Figure 5) [22,23,24]. Virions were enveloped (200–220 nm in diameter) or, more often, non-enveloped (95–105 nm in diameter), with either a stain or non-stain penetrated nucleocapsid. Between the envelope, when present, and the icosahedral capsid, there was an ill-defined, moderately electron-lucent layer (tegument). Capsomers showed deep electron-dense indentations and a typical honeycomb arrangement. No virions were found in the liver and brain tissue samples.

### 3.6. Sequencing and BLAST Results

**Figure 6:**
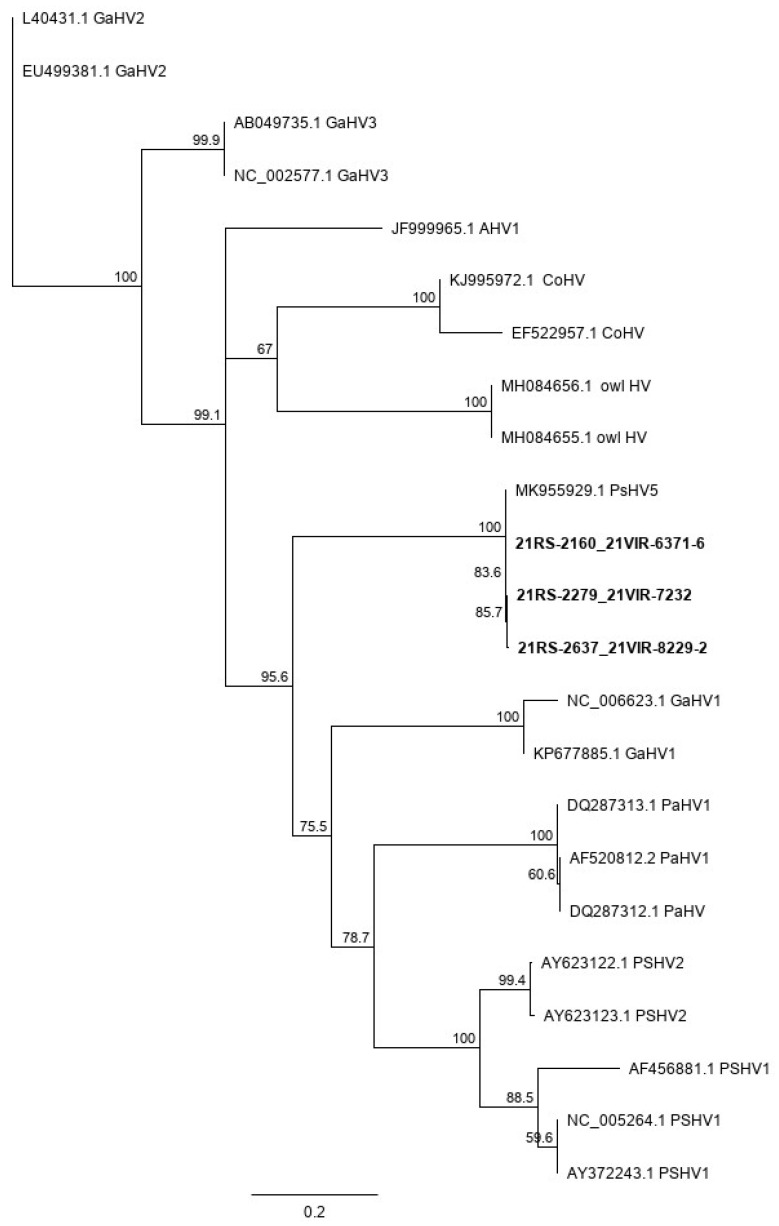
Phylogenetic tree based on the nucleotide sequences of the DNA polymerase gene of avian herpesviruses. The tree was constructed using the maximum likelihood method with 1000 bootstrap replicates and implemented by MEGA X. The PsHV-5 sequences of the present report are identified in bold. The scale bar indicates the number of substitutions per site. Acronym legend: GaHV2 (*Gallid herpesvirus 2*); GaHV3 (*Gallid herpesvirus 3*); AHV1 (*Anatid herpesvirus 1*); CoHV (*Columbid herpesvirus 1*); owlHV (*Strix uralensis herpesvirus*); PSHV5 (*Psittacid herpesvirus 5*); GaHV1 (*Gallid herpesvirus 1*); PaHV (*Passerid herpesvirus 1*); PSHV1 (*Psittacid herpesvirus 1*); PSHV2 (*Psittacid herpesvirus 2*).

The obtained sequences have a percentage identity of 100% exclusively with the only previously identified sequence of PsHV-5 (MK955929.1) [16]. This result confirms what is evidenced by the phylogenetic tree output (Figure 6), since the sequences obtained segregate with the same sequence.

## 4. Discussion

Based on the results of the analyses performed, the authors report the first detection of PsHV-5 in Europe. The authors also reveal that the clinical signs and the gross and histopathological findings associated with PsHV-5 infection are comparable with those previously reported in the literature [16], attesting the tropism of this virus for the respiratory tract. Among the known pathogenic herpesvirus species affecting the birds belonging to the Psittaciformes order, PsHV-3 has shown to have a tropism for the respiratory tract [11,15,25]. It is worth pointing out that other cases of respiratory diseases in parakeets with a herpesvirus as the putative etiology are reported in the literature [8,26]; however, such cases were not investigated at a molecular level, so the etiology remains uncharacterized.

The diagnostic protocol presented in this case report allowed the detection of a virtually unknown pathogen using a non-specific technique on organ samples presenting suspicious lesions. In fact, electron microscopy revealed the presence of viral particles morphologically and dimensionally consistent with herpesvirus, including those previously described in parrots [11,16,27]. In addition, following DNA extraction, amplification and subsequent sequencing of the PCR products, it was finally possible to obtain indications about the presence of PsHV-5 in the sick animals. A primer set targeting the genome region that codifies for the most conserved domain of *Alloherpesviridae* DNA polymerase was used. The obtained sequences, indeed, showed a percentage identity of 100% exclusively with the only previously identified sequence of PsHV-5 (MK955929.1). Therefore, the investigation of the disease agent at a molecular level proved itself essential to achieve a final diagnosis, especially because no specific detection assay is currently available.

Psittacid alphaherpesvirus-5 is a novel virus with an unknown host range. Interestingly, in one of the outbreaks investigated in the present study, the clinical disease was observed in an Alexandrine parakeet bought together with other Indian ringneck parrots. The available literature reports that herpesviruses have likely evolved together with their hosts over time, and for this reason their own phylogenies have mutually conditioned one another [28,29], resulting in a milder form of disease in the “symbiotic” animal host. However, even though this assumption could be applicable to the lower branches of the herpesvirus phylogenetic tree, this may not represent what has happened during the recent evolution of herpesviruses in birds. Indeed, it has been observed that the herpesvirus respiratory clade (GaHV-1, GavHV-1, PasHV-1 and PsHV-3) affects many unrelated host species, and therefore it is more probable that host switching has occurred rather than a co-evolution [11]. This would explain the high morbidity/mortality rates observed in the two related parakeet species involved in the reported outbreaks, although no information is available about the behavior of PsHV-5 in other birds belonging to the Psittaciformes order and, more importantly, in its possible “carrier” species. Since it was not possible to analyze samples from the Alexandrine parakeets, the authors lack information on the viral shedding, the gravity of the histopathological lesions, or tissue tropism in this species, precluding any consideration about the characteristics of the infection in other parrot species.

The outbreaks reported here were characterized by severe clinical signs of respiratory disease. It is known that one of the most common complaints from avian pet owners seeking veterinary advice is respiratory compromise [1,2]. This clinical condition in parrots is of complex medical management, although it is possible to restore the animal’s condition by providing adequate medication associated with prompt supportive care interventions [1]. Therefore, it is fundamental for the pet owner to be aware of the seriousness of the problem in order to reduce the time of medical intervention and to achieve a final diagnosis [30]. Generally, the care that owners show for their pet is greater compared with aviculturists/bird keepers, especially in terms of time spent in close contact with the single animal. In fact, even though pet owners are sometimes less competent in animal ethology and physiology than aviculturists, they can readily recognize abnormal symptomatology and alert their veterinarian. Aviculturists usually keep a high number of different bird species, a situation that leads to a more complex management of bird flocks’ health. It may occur that they do not have the necessary amount of time for a careful assessment of the health status of the aviary, or alternatively, they do not know how to properly carry it out [30,31]. Under these circumstances, it is more likely that the initial signs of sickness will go undetected until a clear deterioration of the disease becomes evident. The result is that anti-contagion interventions within the aviary are implemented when it may already be too late. The present case report describes what was already pointed out by Rasidi and Xie [32], that rapid and efficient communication between pet owners/aviculturists, their veterinarians and pathologists enables achieving early diagnosis and subsequent proper management of the outbreak.

A major critical point for the containment of infectious diseases within the ornamental/exotic bird sector is the dense network and intense interconnections among pet bird owners, bird keepers, breeders and authorized dealers, which lead to an uncontrolled intermingling of animals (e.g., bird fairs/exhibitions) most of the time [33]. This means that there is a high risk of diffusion of infectious agents that can be kept under control by observing the quarantine when introducing new birds into an aviary and, at the same time, by putting in place an early-detection system targeted for disease sign occurrence [30]. To complicate matters, there are infectious agents, such as herpesviruses, which persist in the host for its entire lifetime and that periodically reactivate and produce infectious virus particles that are eventually shed in the environment [4]. The pitfalls of this behavior are that no infectious virus is present during the herpesviral latency period and that the host shows few or no clinical signs, making hard to diagnose the infection. Moreover, both viral reactivation and shedding are not always associated with the disease; therefore, the infection spreads in an even more devious way [34]. During the outbreaks we studied, some parrots never developed any signs of respiratory disease. Unfortunately, it was not possible to test samples of these asymptomatic birds, preventing us from determining if they were shedding the virus or not.

All things considered, the need to disseminate case reports of the disease and how it was diagnosed is tangible, especially because there is no specific assay available for PsHV-5 detection. It is therefore vital to take all the necessary precautions, both in terms of strengthening quarantine measures and, above all, by informing breeders and breeder associations about the circulation of this pathogen. In this way, the diffusion of pathogens within the ornamental bird sector through trade shows and commercial trade may be reduced or avoided, limiting the risk for sensitive species and collections of high genetic value.

## 5. Conclusions

The present report documents the first two diagnosed cases of PsHV-5 infection in Europe, indicating that the geographic distribution of this pathogen is likely broader than what has been observed so far. To the best of the authors’ knowledge, this is just the second detection of this newly described herpesvirus causing respiratory disease in Australian Indian ringneck parrots. Even though there is no specific assay for PsHV-5 detection currently available, the epidemiological surveillance implemented through territorial diagnostic services, together with efficient communication between private practitioners and infectious disease specialists proved themselves fundamental for achieving a definitive diagnosis. Unfortunately, despite what is available for the industrial livestock sector, the applicable oversight procedures for ornamental birds are not evenly developed in the different parts of the world, increasing the number of undiagnosed outbreaks. Therefore, the authors highlight the need to improve both diagnostic services and training of operators of the ornamental bird sector worldwide, with the final aim of preventing uncontrolled pathogen diffusion in this vulnerable sector and of preserving animal health at the same time. In the future, it will be beneficial to develop a specific assay for PsHV-5 detection, which should be added to the currently applied pre- and/or post-movement test panel (Psittacine Beak and Feather Disease virus and Avian Polyomavirus) for parrots.

## Data Availability

Sequence data presented in this case report were deposited in NCBI database with the accession numbers OK665682, OK665683, OK665684.
